# Higher risk of pre-eclampsia and other vascular disorders with artificial cycle for frozen-thawed embryo transfer compared to ovulatory cycle or to fresh embryo transfer following *in vitro* fertilization

**DOI:** 10.3389/fendo.2023.1182148

**Published:** 2023-05-22

**Authors:** Sylvie Epelboin, Julie Labrosse, Jacques De Mouzon, Aviva Devaux, Marie-José Gervoise-Boyer, Laetitia Hesters, Philippe Jonveaux, Rachel Levy, Nathalie Sermondade, Patricia Fauque, Fabienne Pessione

**Affiliations:** ^1^ Department of Reproductive Medicine, Gynecology and Obstetrics, Hôpital Bichat-Claude Bernard, Assistance Publique-Hôpitaux de Paris Nord, Université de Paris, Paris, France; ^2^ Department of Reproductive Medicine, Gynecology and Obstetrics, American Hospital of Paris, Neuilly-sur-Seine, France; ^3^ Department of Reproductive Biology, Unilabs, Direction médicale, Clichy La Garenne, France; ^4^ Department of Reproductive Biology, Centre Hospitalier Universitaire, Amiens, France; ^5^ Department of Reproductive Medicine, Hôpital Saint Joseph, Marseille, France; ^6^ Department of Reproductive Biology, Hôpital Antoine Béclère, Hôpitaux Universitaires Paris-Sud, Assistance Publique - Hôpitaux de Paris, Clamart, France; ^7^ Department of Procreation, Agence de la biomédecine, La Plaine Saint Denis, France; ^8^ Department of Reproductive Biology, Hôpital Tenon, Hôpitaux Universitaires Est Parisien, Assistance Publique – Hôpitaux de Paris, Paris, France; ^9^ Department of Reproductive Biology, Université Bourgogne Franche-Comté - INSERM UMR1231, Dijon, France

**Keywords:** pre-eclampsia, artificial cycle, ovulatory cycle, frozen embryo transfer, fresh embryo transfer, endometrial preparation

## Abstract

**Background:**

Risks of maternal morbidity are known to be reduced in pregnancies resulting from frozen embryo transfer (FET) compared to fresh-embryo transfer (*fresh*-ET), except for the risk of pre-eclampsia, reported to be higher in FET pregnancies compared to *fresh*-ET or natural conception. Few studies have compared the risk of maternal vascular morbidities according to endometrial preparation for FET, either with ovulatory cycle (OC-FET) or artificial cycle (AC-FET). Furthermore, maternal pre-eclampsia could be associated with subsequent vascular disorders in the offspring.

**Methods:**

A 2013-2018 French nationwide cohort study comparing maternal vascular morbidities in 3 groups of single pregnancies was conducted: FET with either OC or AC preparation, and *fresh*-ET. Data were extracted from the French National Health System database. Results were adjusted for maternal characteristics and infertility (age, parity, smoking, obesity, history of diabetes or hypertension, endometriosis, polycystic ovary syndrome and premature ovarian insufficiency).

**Results:**

A total of 68025 single deliveries were included: *fresh*-ET (n=48152), OC-FET (n=9500), AC-FET (n=10373). The risk of pre-eclampsia was higher in AC-FET compared to OC-FET and *fresh*-ET groups in univariate analysis (5.3% *vs.* 2.3% and 2.4%, respectively, *P*<0.0001). In multivariate analysis the risk was significantly higher in AC-FET compared to *fresh*-ET: aOR=2.43 [2.18-2.70], *P*<0.0001). Similar results were observed for the risk of other vascular disorders in univariate analysis (4.7% *vs*. 3.4% and 3.3%, respectively, *P*=0.0002) and in multivariate analysis (AC-FET compared to *fresh*-ET: aOR=1.50 [1.36-1.67], *P*<0.0001). In multivariate analysis, the risk of pre-eclampsia and other vascular disorders were comparable in OC-FET and *fresh*-ET: aOR=1.01 [0.87-1.17, *P*= 0.91 and aOR=1.00 [0.89-1.13], *P*=0.97, respectively).Within the group of FET, the risks of pre-eclampsia and other vascular disorders in multivariate analysis were higher in AC-FET compared to OC-FET (aOR=2.43 [2.18-2.70], *P*<0.0001 and aOR=1.5 [1.36-1.67], *P*<0.0001, respectively).

**Conclusion:**

This nationwide register-based cohort study highlights the possibly deleterious role of prolonged doses of exogenous estrogen-progesterone supplementation on gestational vascular pathologies and the protective role of the *corpus luteum* present in OC-FET for their prevention. Since OC-FET has been demonstrated not to strain the chances of pregnancy, OC preparation should be advocated as first-line preparation in FET as often as possible in ovulatory women.

## 1 Introduction

The practice of frozen-thawed embryo transfer (FET) has increased over the past decades in connection with technological improvements resulting in higher cumulative live birth rates (1–5). FET enables single embryo transfer, reduces the risk of ovarian hyperstimulation syndrome, optimizes endometrial receptivity and facilitates fertility preservation (2–8). According to the 2020 annual report of the French Biomedicine Agency, FET was performed in up to 33 350 couples, representing 41.6% of *in vitro* Fertilization (IVF) transfers, of which 65% were transfers of frozen-thawed blastocysts. Although there is no transnational report on the type of protocol most frequently used for FET, it seems that the artificial cycle (AC) is the most performed worldwide since it facilitates the organization of ART centers compared with endometrial preparation by ovulatory cycles (OC), whether natural/modified natural or stimulated.

Assisted Reproductive Technologies (ART) have been associated to various maternal morbidities for which controlled ovarian stimulation regimens (9), embryo culture media (10, 11), and/or subfertility in itself might play a role (12–14). Risks of maternal and perinatal morbidity (placenta previa, placental abruption, premature birth, small for gestational age, and perinatal mortality) are known to be reduced in pregnancies resulting from FET compared to fresh embryo transfer (*fresh*-ET), except for the risk of pre-eclampsia and severe pre-eclampsia that were reported to be significantly higher in pregnancies resulting from FET compared to *fresh*-ET or natural conception (9, 15–21). Gestational hypertension is a disease of pregnancy that combines an increase in blood pressure > 140 mmHg and/or 90 mmHg occurring after the 20th week of amenorrhea, measured twice, with or without proteinuria > 0.3g/24h and/or oedemas and clinical other symptoms (22). The severe form of pre-eclampsia is defined by systolic pressure is ≥ 160 mmHg and/or diastolic pressure ≥ 110 mmHg or hypertension not controlled by treatment, more or less associated with impaired renal function with proteinuria ≥ 3g per 24 hours, oliguria, high blood levels of creatinine and/or liver enzymes, and low levels of blood platelets (22). Preeclampsia can lead to eclampsia, a serious complication that results in seizures.

Pre-eclampsia is considered as a multi-systemic disorder occurring in about 2-5% of pregnancies and as one of the leading causes of maternal and perinatal mortality and morbidity, particularly in developing countries (23). In France, although maternal mortality secondary to hypertensive pathologies in pregnancy has decreased by 50% in 10 years according to the latest report of the confidential national survey on maternal deaths, pre-eclampsia and severe pre-eclampsia remains one of the main causes of mortality (24). Moreover, beyond maternal mortality, pre-eclampsia can be the cause of significant severe maternal morbidity: in 10% of cases, pre-eclampsia progresses to a severe form, which can lead to organic dysfunctions, sometimes persistent in the medium and long term. Severe pre-eclampsia is a risk factor for postpartum thromboembolic complications. Pre-eclampsia is also responsible for a third of premature births in France (25). Therefore, it is important to prevent it by refining the knowledge of risk factors. An increased risk of pre-eclampsia might be explained by altered trophoblastic invasion leading to an inadequate remodelling of spiral arteries, insufficiently dilated vessels, an imbalance between angiogenic and anti-angiogenic factors or by endothelial dysfunction occurring after the release of placental factors into maternal circulation (26). Some causes of female infertility, such as diminished ovarian reserve or endometriosis may be a risk factor for pre-eclampsia and placental malperfusion lesions (27, 28).

Concerning the impact of the different types of endometrial preparation protocols for FET on the risk of pregnancy-induced vascular disorders, some studies have suggested that the presence of a *corpus luteum* (CL) in OC might be a protective factor, whereas the non-physiological and prolonged doses of the estrogen-progesterone combination in AC may have a deleterious impact (20, 29).Thus, as most recent studies demonstrate an equal live birth rate with endometrial preparation either by OC or AC, others have compared the risk of gestational vascular morbidities between the two types of protocols (20, 29). A systematic review of 2021 on hypertensive disorders in pregnancy following a FET cycle observed that hypertensive disorders were significantly increased after AC-FET when compared with natural cycle or mild OC-FET cycle (30). In a 2022 meta-analysis of 12 studies, Busneli et al. (31) observed a higher risk of pre-eclampsia, hypertensive disorders of pregnancy (HDP) and pregnancy-induced hypertension in AC-FET pregnancies compared to NC.

Many studies have also highlighted other manifestations of gestational vascular disorders, not only on pregnancy, but on the child’s growth, in case of assisted procreation techniques or treatments (30–35). Nevertheless, we have chosen to focus this study on the association between endometrial preparation protocols for ET and the incidence of pre-eclampsia and other maternal vascular disorders during pregnancy. The study of fetal growth disorders following medically assisted reproduction according to maternal context or techniques was specifically studied from the same national cohort by the same research group from the French Biomedecine Agency.

The objective of this extensive 5-year nationwide register-based cohort study was to evaluate, in the French population, the risk of pre-eclampsia and other gestational vascular disorders between FET, according to endometrial preparation by either ovulatory cycle (OC-FET) or artificial cycle (AC-FET) and *fresh*-ET. The aim was to report broad real-life data considering the numerous confounding factors accessible in the database, including underlying female infertility.

## 2 Materials and methods

This study is a nationwide register-based cohort study. Data were extracted from the French National Health System database (*Système National des Données de Santé – SND*S) that includes > 99% of national deliveries, in which all outpatients and hospitalizations from 2008 to 2018 (in any public hospital and private clinic) were registered. The database contains information on patient characteristics, diagnoses and treatments registered in outpatient consultations. Maternal records were merged anonymously and with previous hospitalisations through a specific software making it impossible to retrieve patient identity but allowing to cross information through anonymized codes. The access to this database was legally approved.

We conducted a comparative analysis of the cohort of singleton births (deliveries ≥22 weeks of gestation (WG) and/or > 500g of birthweight) occurring in France and resulting from *fresh*-ET or FET from IVF and intracytoplasmic sperm injection (ICSI) cycles performed over a 5-year period (2013-2017). All women with a history of delivery from IVF with fresh-ET, intrauterine insemination or FET within the previous 5 years were excluded from the analysis As it was specified above, the database analysis made it possible to identify health events of our patients since the year 2008. Data available in the hospitalization database were parity, multiple pregnancy, maternal age, active smoking during pregnancy, obesity, maternal history of diabetes (type 1 or 2) or hypertension, diagnosis of endometriosis, polycystic ovary syndrome (PCOS) or premature ovarian insufficiency (POI), mode of conception (*fresh*-ET or FET) and term. Patients with twin deliveries or history of hypertensive disorders (pregnancy-induced or not) were excluded.

### 2.1 Comparison groups

Three comparison groups of singletons were analyzed: 1/pregnancies resulting from OC-FET (natural, modified natural or stimulated cycle); 2/pregnancies resulting from AC-FET; 3/pregnancies resulting from *fresh*-ET.

### 2.2 Endometrial preparation protocols

Endometrial preparation with OC included natural cycles, modified natural cycles (natural cycles with ovulation triggering by hCG and/or luteal phase support) and stimulated cycles (mild ovarian stimulation by gonadotropins). Luteal phase support with vaginal micronized progesterone (VMPg, 200 to 400 mg/day) was administered for 6 to 10 weeks of gestation (WG) in case of pregnancy.

Endometrial preparation by AC consisted in the sequential administration of exogenous estrogens and progesterone. According to ART centers, supplementation by estrogens was usually started on Day 1 (orally at 4-8 mg/day and/or transdermally at 200 µg/3days). In case of AC protocols with previous down regulation by GnRH-agonist, estrogen supplementation was started 10 to 15 days after GnRH-agonist introduction. Once adequate endometrial thickness was obtained, VMPg (600 to 1200 mg daily) was started and continued until the 12th WG in case of pregnancy.

Embryos were obtained from conventional IVF or ICSI cycles. In FET groups, embryos were frozen at cleavage stage or blastocyst stage (according to the evolution of laboratory policies).

### 2.3 Vascular disorders

Vascular disorders were classified into 2 groups according to the International Classification of Disease (ICD-10) codes: (i) hospitalization for pre-eclampsia and/or eclampsia (O14 and O15, grouped under the term pre-eclampsia), and (ii) hospitalization for other vascular disorders during pregnancy (gestational hypertension with or without proteinuria (O13, O16) or isolated proteinuria with oedema (O12). Although the terms used in literature are most often hypertensive disorders of pregnancy, (grouping pre-eclampsia and pregnancy induced hypertension with or without proteinuria), we made the choice to scrupulously follow the diagnoses according to the ICD10 codes internationally validated. We grouped under the term “other vascular disorders” the cases of hospitalization for isolated proteinuria with oedema with those for gestational hypertension with or without proteinuria, and distinguished them from pre-eclampsia/eclampsia, because of the difference in the severity of the maternal-fetal consequences. For isolated proteinuria with oedema, we make it clear that these are cases that have generated hospitalization.

### 2.4 Statistical analysis

Univariate and multivariate analyses were performed using logistic regression models to compare the risk of pregnancy-induced vascular disorders between *fresh*-ET and FET, and between the two types of endometrial preparation in FET cycles (OC-FET *versus* AC-FET). Two risk estimations were performed in multivariate analysis: the risk of pre-eclampsia and the risk of other pregnancy-induced vascular disorders, compared to pregnancies without any vascular disorder. Adjusted Odds Ratios (aOR) and their 95% confidence intervals (CI) were estimated.

## 3 Results

The study included 68 025 single deliveries following embryo transfer occurring nationwide from 2013 to 2017. A total of 48 152 were cases of *fresh*-ET and 19 873 were FET cycles, among which 9 500 were AC-FET and 10 373 were OC-FET.

### 3.1 Maternal characteristics

Comparison of maternal characteristics according to the type of treatment is presented in **Table 1**. Patients with *fresh*-ET were younger and more often primiparous (*P* < 0.0001 in the overall comparison). Within FET groups, in univariate analysis, women with AC were more often primiparous (56.1% *vs.* 54.5%, *P* = 0.03) and obese (4.3% *vs.* 3.6%, *P* =0.01) and were more often diagnosed with endometriosis (13.3% *vs.* 10.8%, *P* < 0.0001), PCOS (3.5% *vs.* 2.0%, *P* < 0.0001) and POI (1.4% *vs.* 0.6%, *P* < 0.0001).

**Table 1:** Maternal characteristics according type of embryo transfer and endometrial preparation protocol.

	*Fresh*-ET N=48152		FET without AC N=9500		FET with AC N=10373		P*
	N	%	N	%	N	%	
**Age (years)**	33.2	4.4	33.4	4.2	33.5	4.3	
30-29	10101	21.0	1755	18.5	1948	18.8	< 0.0001
30-39	34080	70.8	6980	73.5	7486	72.2	
≥ 40	3971	8.3	765	8.1	939	9.1	
**Primiparous**	31221	64.8	5181	54.5	5816	56.1	< 0.0001
**Smoking**	1048	2.2	163	1.7	211	2.0	0.02
**Obesity BMI > 30**	1818	3.8	340	3.6	445	4.3	0.02
**Diabetes**	365	0.8	63	0.7	83	0.8	0.51
**Endometriosis**	6080	12.6	1025	10.8	1374	13.3	< 0.0001
**PCOS**	1077	2.2	185	2.0	362	3.5	< 0.0001
**POI**	676	1.4	60	0.6	141	1.4	< 0.0001
**Pregnancy-induced vascular disorders**							
Pre-eclampsia	1162	2.4	214	2.3	546	5.3	< 0.0001
Other vascular disorders	1621	3.4	309	3.3	486	4.7	< 0.0001

Fresh-ET, fresh embryo transfer; FET, frozen-thawed embryo transfer; AC, artificial cycle; BMI, body mass index; PCOS, polycystic ovarian syndrome; POI, premature ovarian insufficiency.

*P-values in the overall comparison.

### 3.2 Risk of pre-eclampsia and other vascular disorders

The frequency of hospitalizations for pre-eclampsia was 2.8% (n = 1 922) and 3.6% (n = 2 416) for other vascular disorders. A total of 63 687 (93.6%) women were not hospitalized for any vascular disorder.

Risk factors of pre-eclampsia and other vascular disorders compared to women without any vascular disorder in multivariate analysis are presented in **Tables 2**, **3**, respectively. The risk of pre-eclampsia increased with age, primiparity, obesity, history of diabetes and POI (**Table 2**). There was no increased risk of pre-eclampsia in case of endometriosis or PCOS. The risk of vascular disorders other than pre-eclampsia increased with age, active smoking, primiparity, obesity and history of diabetes (**Table 3**). There was no increased risk based on any maternal infertility.

**Table 2:** Risk of pre-eclampsia compared to no vascular disease in multivariate analysis (N=65609).

	Risk of pre-eclampsia*					
	N	%	Adjusted OR	CI 95%		*P*
**All**	1922	2.9				
Maternal age						
20-29	360	2.6	1			
30-39	1332	2.7	1.19	1.05	1.34	0.005
≥ 40	230	4.1	1.76	1.49	2.09	< 0.0001
Smoking						
No	1879	2.8	1			
Yes	43	3.0	1.02	0.45	1.40	0.88
Primiparous						
No	467	1.8	1			
Yes	1455	3.5	2.13	1.91	2.37	< 0.0001
Obesity						
No	1742	2.7	1			
Yes	180	6.9	2.72	2.31	3.20	< 0.0001
Diabetes						
No	1881	2.8	1			
Yes	41	8.0	2.97	2.13	4.14	< 0.0001
Endometriosis						
No	1684	2.8	1			
Yes	238	2.8	0.97	0.45	1.12	0.71
PCOS						
No	1870	2.8				
Yes	52	3.2	0.99	0.45	1.32	0.97
POI						
No	1876	2.8	1			
Yes	46	5.3	1.77	1.31	2.40	0.0002
Treatment						
*Fresh*-ET (N=46531)	1162	2.5	1			
FET without AC (N=9191)	214	2.3	1.01	0.87	1.17	0.91
FET with AC (N=9887)	546	5.5	2.43	2.18	2.70	< 0.0001

Pre-eclampsia/eclampsia is defined according to the International Classification of Disease (ICD-10) codes: (O14 and O15).

Patients with other vascular diseases are excluded: N =65609: 1922 with preeclampsia vs. 63687 without any vascular disease.

Fresh-ET, fresh embryo transfer; FET, frozen-thawed embryo transfer; AC, artificial cycle; confidence intervals; aOR, adjusted odds ratio; PCOS, polycystic ovarian syndrome; POI, premature ovarian insufficiency.

**Table 3:** Risk of other pregnancy-induced vascular diseases compared to no vascular disease in multivariate analysis (N =66103).

	Risk of other vascular disorders					
	N	%	Adjusted OR	CI95%		*P*
**All**	2416	3.7				
Maternal age						
20-29	477	3.6	1			
30-39	1691	3.6	1.07	0.96	1.19	0.20
≥ 40	248	4.6	1.37	1.17	1.61	< 0.0001
Smoking						
No	2348	3.6	1			
Yes	68	4.9	1.36	1.06	1.74	0.02
Primiparous						
No	774	3.1	1			
Yes	1642	4.0	1.3	1.26	1.51	< 0.0001
Obesity						
No	2276	3.6	1			
Yes	140	5.8	1.60	1.34	1.91	< 0.0001
Diabetes						
No	2370	3.6	1			
Yes	46	9.8	2.72	1.99	3.71	< 0.0001
Endometriosis						
No	2107	3.6	1			
Yes	309	3.8	1.02	0.90	1.15	0.73
PCOS						
No	2349	3.6	1			
Yes	67	4.3	1.11	0.87	1.43	0.40
POI						
No	2384	3.7	1			
Yes	32	3.9	1.02	0.71	1.45	0.93
Treatment						
*Fresh*-ET (N=46990)	1621	3.4	1			
FET without AC (N=9286)	309	3.3	1.00	0.89	1.13	0.98
FET with AC (N=9827)	486	5.0	1.50	1.36	1.67	< 0.0001

Other vascular disorders are defined according to the International Classification of Disease codes: hospitalization for (gestational hypertension with or without proteinuria (O13, O16) or isolated proteinuria with oedema (O12).

Patients with preeclampsia are excluded: N=66103: 2416 other vascular disease and 63687 without any vascular disease.

Fresh-ET, fresh embryo transfer; FET, frozen-thawed embryo transfer; AC, artificial cycle; CI, confidence intervals; aOR, adjusted odds ratio; PCOS, polycystic ovarian syndrome; POI: premature ovarian insufficiency.

The risk of pre-eclampsia was significantly higher in the AC-FET group compared to OC-FET and *fresh*-ET groups in univariate analysis (5.3% *vs.* 2.3% and 2.4%, respectively, *P* < 0.0001). Similar results were observed for the risk of other vascular disorders in AC-FET compared to OC-FET and *fresh*-ET in univariate analysis (4.7% *vs.* 3.4% and 3.3%, *P* = 0.0002) (**Table 1**).

In multivariate analysis, the risk of pre-eclampsia compared to no vascular disease was significantly higher in AC-FET compared to *fresh*-ET (aOR = 2.43 [2.18-2.70], *P* < 0.0001) (**Table 2**). The risk was similar between OC-FET and *fresh*-ET: (aOR = 1.01 [0.87-1.17], *P* = 0.91) (**Table 2**).

In multivariate analysis, the risk of other vascular disease compared to no vascular disease was significantly higher in AC-FET compared to *fresh*-ET (aOR = 1.50 [1.36-1.67], *P* < 0.0001) (**Table 3**). The risk was similar between OC-FET and *fresh*-ET (aOR = 1.00 [0.89-1.13], *P* = 0.97, respectively) (**Table 3**).

**Figure 1:**
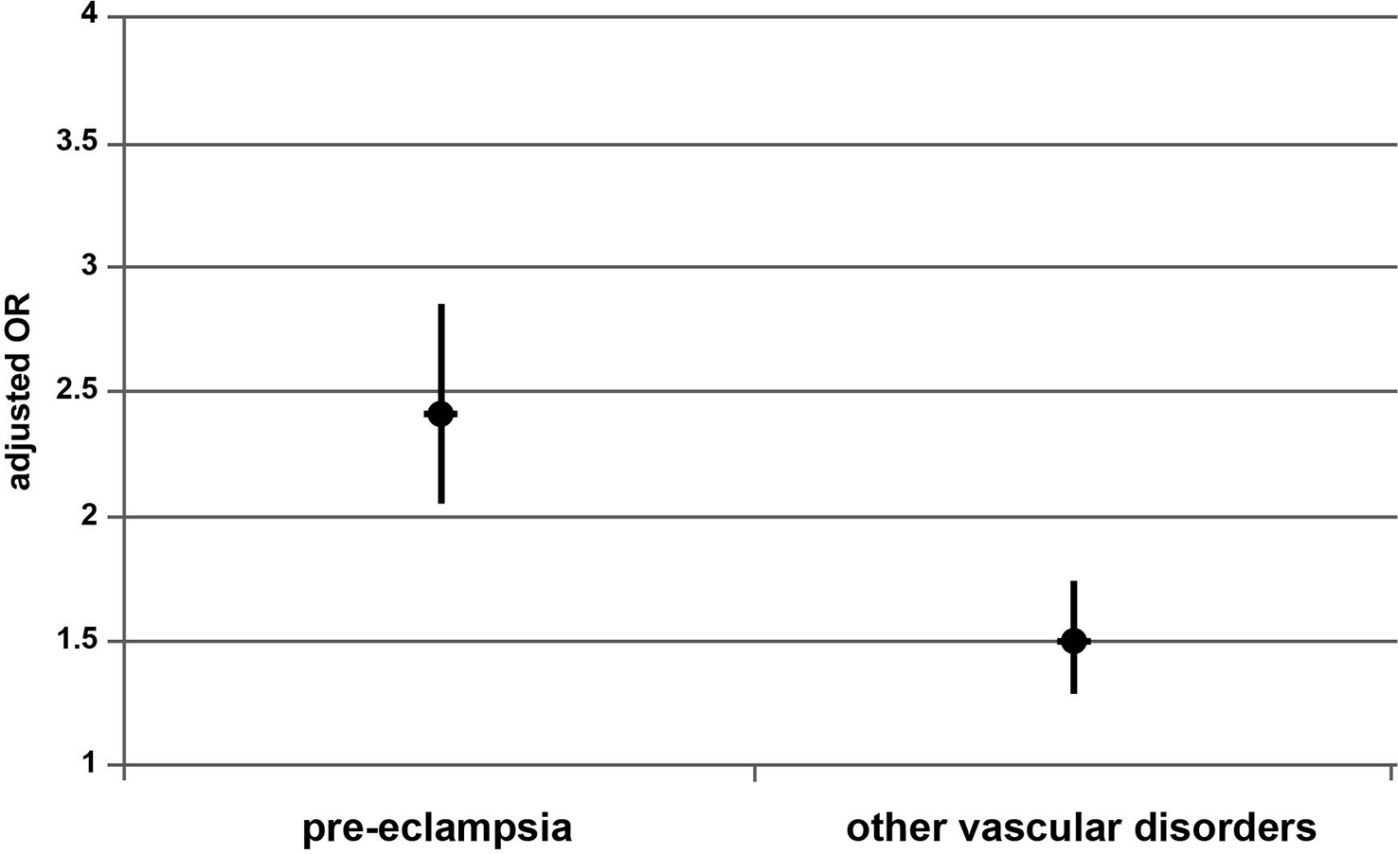
Risk of pre-eclampsia and other vascular disorders after AC-FET compared to OC-FET. AC-FET, artificial cycle for frozen-embryo transfer; OC-FET (line 1), ovulatory cycle for frozen-embryo transfer.

To confirm the difference between the two endometrial preparation protocols for FET, the same multivariate analyses were performed excluding patients with *fresh*-ET. The risk of pre-eclampsia and other vascular disorders (compared to no vascular disease) was significantly higher after AC-FET compared to OC-FET: aOR=2.42 [2.06-2.85], *P* < 0.0001 and aOR=1.50 [1.29-1.74], *P* < 0.0001, respectively) (**Figure 1**).

## 4 Discussion

In all, the findings from this large 5-year nationwide observational study demonstrated that endometrial preparation by AC was associated with an increased risk of pre-eclampsia and other vascular disorders compared to *fresh*-ET and OC-FET. These results remained significant after multivariate analysis adjusted on maternal characteristics. The risk was similar between OC-FET and *fresh*-ET.

Several recent publications are in line with our findings. A 2022 retrospective cohort study analysing the incidence of pre-eclampsia in 536 pregnant patients (from either autologous cycles or egg donation) after OC-FET (n = 325) or AC-FET (n= 211) showed that pre-eclampsia was significantly higher in AC cycles (11.8% *vs*. 3.7%, respectively, *P* < 0.001). Results remained significant after multivariate logistic regression analysis (AC-FET *vs.* OC-FET: OR: 2.9, 95% CI 1.4–6.0, *P* = 0.005) (36). Similarly, a 2022 meta-analysis including 9 studies (n= 8 327 patients with PCOS, pregnant after AC-FET or OC-FET) showed that preterm birth and pre-eclampsia rates were significantly higher with AC-FET compared to OC-FET (37). In addition to the obstetrical aspect, long-term consequences of pre-eclampsia are becoming a concern. A 2021 population-based cohort study using Danish national health registers including 2 491 340 individuals born in Denmark from 1977 to 2018 suggest that the existence of maternal hypertensive disorders during pregnancy is associated with a 23% increased risk of early-onset cardiovascular disease in children (32). Notably, results show an increased risk of specific cardiovascular diseases such as hypertension (HR = 2.11 [1.96-2.27]; *P* < 0.001), myocardial infarction (HR = 1.49 [1.12-1.98]; *P* = 0.007), pulmonary embolism (HR = 1.33 [1.11-1.58]; *P* = 0.002) and heart failure (HR = 1.30 [1.02-1.66]; *P* = 0.037). Moreover, a 2022 multinational population-based cohort study collecting data from Danish, Finnish and Swedish national registries (including 8 475 819 births, of which 188 670 (2.2%) were exposed to maternal pre-eclampsia) showed that children had an increased risk of ischemic heart disease (aOR = 1.33 [1.12-1.58]) and stroke (aOR = 1.34 [1.17-1.52]) in case of maternal pre-eclampsia (38). These associations were independent from preterm or small for gestational age, but dependent on the severity of pre-eclampsia.

In line with our findings, recent studies describe increased obstetrical risks in the absence of CL (18). The pathophysiology of vascular disorders found increased in the absence of CL seems to involve multiple factors. Indeed, the CL is a major source of estradiol, progesterone and their metabolites, as well as relaxin and vasoactive and angiogenic substances that might optimize implantation and placentation. Therefore, the presence of a CL in endometrial preparation by OC possibly leads to more physiological protein secretion profiles compared to AC (39, 40). A hypothesis for the increased risk of pre-eclampsia in the absence of CL could be the imbalance of steroid hormones and their metabolites influencing early physiological processes such as decidualisation, implantation, angiogenesis and maternal haemodynamic (41). Moreover, serum relaxin levels are almost undetectable in pregnant women without CL. This absence of circulating relaxin may also be at risk of abnormal placentation or compromised maternal cardiovascular adaptation. In 2019, Von Versen-Höynck et al. prospectively assessed rates of gestational vascular pathologies in relation to carotid-femoral pulse wave velocity and transit time before, during and after pregnancy, according to number of CL: 0 (n = 26), 1 (n = 23) or >1 (n = 22) (42). AC-FET cycles (0-CL) were associated with higher rates of pre-eclampsia (12.8% *vs*. 3.9%, *P* = 0.02) and severe pre-eclampsia (9.6% *vs*. 0.8%, *P* = 0.002) compared to modified natural FET cycles (1 CL). Authors suggested that altered vascular health in early pregnancy in women with 0 CL (AC cycles) might lead to insufficient cardiovascular adaptation contributing to an increased risk of pre-eclampsia (43). Moreover, within the same time, Boutet et al. published a case-control study on maternal and fetal concentrations of haemopexin, a glycoprotein protective of the vascular endothelium, in pre-eclamptic IVF-pregnancies according to presence or not of CL at embryo transfer (44). After adjustment, maternal haemopexin was higher in IVF with CL compared to natural conception in normotensive women (*P* = 0.04) and in case of pre-eclampsia (*P* = 0.01), and lower in case of pre-eclampsia in IVF pregnancies without CL compared to IVF pregnancies with CL (*P* = 0.002). In cord blood, in case of pre-eclampsia, hemopexin was higher in IVF with CL when compared to spontaneous pregnancies (*P* = 0.04). These physiological differences support the hypothesis that CL activity may influence perinatal outcomes.

The strength of our extensive 5-year nationwide register-based cohort study covering 68025 single deliveries including 19873 resulting from FET, almost equally between preparation by artificial (AC-FET: 10373) or ovulatory cycle (OC-FET: 9500), and 48152 fresh-ET controls, relies in the number and exhaustiveness of subjects analyzed. Moreover, our national database allows us to report broad real-life data considering the numerous confounding factors accessible in the database, including underlying detailed female infertility.

The limitations are linked to the register-based nature of the cohort data, which, although collected prospectively for the National Health Data System, were analyzed retrospectively according to a non-predetermined reading protocol. Therefore, it did not enable to refine the risk according to details of techniques (embryo stage, culture media, slow freezing or vitrification) and treatments (such as use or not of antiplatelet agents) in each group. Our national database provides some information on certain maternal characteristics (age, obesity, POI, PCOS, endometriosis, etc…) but does not enable to establish a possible link with the indication of FET (avoid fresh transfer to prevent the risk of OHSS, deferred transfer of supernumerary embryo after failure of fresh transfer or after a previous pregnancy). Nevertheless, except for POI, maternal underlying infertility (including PCOS) did not impact the incidence of gestational vascular disorders when comparing fresh-ET and both FET populations (**Table 2**). Therewith, we did not investigate the respective number of deliveries for the same mother over the period studied, which could result either from two successive IVFs with fresh-transfer if there are no supernumerary embryos after the first delivery, or FET for another child after successful IVF and fresh-transfer delivery, or FET for a child followed by IVF with fresh-transfer for the next one, or again2 deliveries after FET. The hypothesis of an increased risk of gestational vascular pathologies in the event of a prior history of gestational hypertensive disorder could be relevant. Nevertheless, the interpretation would be complex since a vascular gestational history induces a preventive therapeutic framework for the subsequent pregnancy, and because of the multiple possible successions of protocols. In this study, except for ET protocols, the risk of pre-eclampsia and other vascular disorders also increased with age, primiparity, obesity, history of diabetes and POI. Given the multicentric and retrospective nature of this national cohort, it was not possible for us to test the hypothesis of any association between those factors and the protocol choose for ET

In the growing trend of ART centers to practice more and more embryonic freeze-all, the choice of endometrial preparation in full knowledge of its side effects is of primary importance. The two lessons learned from these broad national data are as much warning information regarding the risk factor for preeclampsia and other vascular disorders represented by the artificial cycle preparation, than a reassuring message stemming from the data concerning the ovulatory cycle.

The choice of treatment for FET is possible in women whose ovarian function is present. Growing evidence highlights the possibly fundamental nature of the contribution of the CL in the prevention of adverse obstetrical and perinatal outcomes during pregnancy (45). Pereira et al. (41) stated that a better understanding of the critical roles of the secretory products of the CL during early pregnancy held the promise of improving the efficacy and safety of ART based on programmed FET cycles.

Until recently, the decision on the endometrial preparation protocol most often depended on center procedures, based on medical arguments, expected success rates, pregnancy loss rates, feasibility, organization and regulation of the center’s activity and women’s comfort. Medical arguments are essentially based on the possibility of ovulation of the woman. Obviously, dysovulations or anovulations determine a preparation with AC. If AC cycle with or without GnRH-agonist pre-treatment has long been the first choice for PCOS patients, current trends follow the principles of individualization, securitization and optimization in endometrial preparation, as mean endometrial thickness, implantation rates, clinical pregnancy rates, ongoing pregnancy rates and live birth rates are similar in artificial cycle and stimulated cycle for endometrial preparation prior to FET in PCOS (46, 47). Guo et al’s recent retrospective study on 1413 cases suggested that natural cycle, hormone replacement cycle, or hormone replacement treatment with GnRHa pretreatment showed no superiority or inferiority in pregnancy and perinatal outcomes in patients with endometriosis (48). Furthermore, the organizational argument has long prevailed in favor of AC-FET, which makes it possible to regulate transfers along the week. However, the use of antagonists in modified natural cycles allows an almost comparable flexibility. The argument of women’s comfort between a few days of subcutaneous injections in the stimulated protocols then about 6 weeks of progesterone (the vaginal route being the most used in France) in OC, and 3 months of oral estrogen intake and vaginal progesterone in AC protocol, is a very subjective consideration. The cost-effectiveness argument between the two protocols, including the cost of pregnancy concerns (such as hospitalization for pregnancy loss or pre-eclampsia and its complications), has poorly been studied and varies according to the country and the financial support. A recent retrospective study considering overweight/obese women with PCOS, suggested that midly stimulated preparation for FET demonstrated a higher LBR and a lower pregnancy loss rate than that in the AC-FET, and may be considered. as the most cost-effective treatment with the least adverse effects on patients (49). The argument for success has long been in favor of AC-FET, until considering the increased number of miscarriages and therefore the lower live birth rate (LBR) with AC when compared with OC.”

Zhang et al. (37) suggested that endometrial preparation by OC might be superior to AC, with significantly higher live birth rates and lower risks of miscarriage, preterm birth and pre-eclampsia, even for women with PCOS (37). Von Versen-Höynck et al. also concluded that pregnancy in the absence of CL could lead to adverse maternal and foetal risks and suggested that the existing evidence was already sufficient to discourage the use of AC-FET in women who ovulate, as they generate a deviation from physiology, exposing the patient and fetus to an avoidable health risk with no apparent benefit (42).

Hence, wider implications of this nationwide register-based cohort study are that it highlights two important information for physicians: i) the possible deleterious role of non-physiological and prolonged doses of exogenous estrogen-progesterone supplementation on gestational vascular pathologies ii) the protective role of the CL present in stimulated or spontaneous OC for their prevention. Our conclusions could help to change habits, especially since the possible addition of antagonists in OC allows a satisfactory programming of embryo transfer, which is the principal advantage of using AC in ART centers. Since results obtained by OC do not strain the chances of pregnancy, OC preparation could be advocated as first-line endometrial preparation in FET as often as the choice is possible in ovulatory women. Nevertheless, one must consider what specific management could be proposed for cases in which AC is unavoidable, as egg donation for pre-menopausal or menopausal women, or irreducible cases of anovulation or dysovulation generating a long and painful stimulation in women. Developing strategies to reduce the risk of pre-eclampsia are required. The preventive efficacy of antiplatelet agents in AC remains to be established. The present study should also be extended by a long-term follow-up of the cohort in order to evaluate the possible association of maternal pre-eclampsia with an increased risk of subsequent vascular pathologies in the offspring.

## Data availability statement

The datasets presented in this article are not readily available because we used the French National hospitalization database (PMSI), included in the large French National Health System database (Système National des Données de Santé (SNDS), in which all hospitalizations (in any public hospital or private clinic) are registered, containing information on patient characteristics, diagnoses and treatments. Data were anonymized at data entry through a specific software making it impossible to retrieve patient identity but enabling to follow all hospitalizations through anonymized codes. Access to PMSI data and SNDS was legally approved in accordance with French Public Health Law (decree N° 2016-1871). Access to PMSI and SNDS data for organizations that do not have permanent access or matching with other databases already available goes through an authorization procedure that involves several organizations: the National Data Institute health (INDS, which in 2019 became the health data platform, the Expertise Committee for research, studies and evaluations in the field of health (CESREES). Consequently, data are available after obtaining legal authorization (at https://www.indsante.fr/) and from the CNIL (Commission Nationale Informatique et Liberte; CNIL, https://www.cnil.fr/). According to the French Public Health Law, non-interventional studies on humans do not require approval from an Institutional Review Board or written consent from participants. The study was conducted according to institutional and ethical rules concerning research on tissue specimens and patients. Requests to access the datasets should be directed to sylvie.epelboin@aphp.fr.

## Ethics statement

Ethical review and approval were not required for the study on human participants in accordance with the local legislation and institutional requirements. Written informed consent for participation was not required for this study in accordance with the national legislation and the institutional requirements.

## Author contributions

SE, FP: study conception and design. SE, JL, PF, FP: methodology and investigation. FP: formal analysis. JM, M-JG-B, LH, NS, RL, PJ: resources. SE, JL, PF, FP: writing—original draft preparation. SE, JL, PF, FP: writing—review and editing. All authors contributed to the article and approved the submitted version.
